# Insights into How Nucleotide-Binding Domains Power ABC Transport

**DOI:** 10.1016/j.str.2009.07.009

**Published:** 2009-09-09

**Authors:** Simon Newstead, Philip W. Fowler, Paul Bilton, Elisabeth P. Carpenter, Peter J. Sadler, Dominic J. Campopiano, Mark S.P. Sansom, So Iwata

**Affiliations:** 1 Division of Molecular Biosciences, Membrane Protein Crystallography Group, Imperial College London, Exhibition Road, London SW7 2AZ, UK; 2 Department of Biochemistry, University of Oxford, South Parks Road, Oxford OX1 3QU, UK; 3 School of Chemistry, University of Edinburgh, Edinburgh EH9 3JJ, UK; 4 Membrane Protein Laboratory, Diamond Light Source Ltd, Harwell Science and Innovation Campus, Chilton, Didcot OX11 ODE, UK

**Keywords:** CELLBIO, PROTEINS

## Abstract

The mechanism by which nucleotide-binding domains (NBDs) of ABC transporters power the transport of substrates across cell membranes is currently unclear. Here we report the crystal structure of an NBD, FbpC, from the *Neisseria gonorrhoeae* ferric iron uptake transporter with an unusual and substantial domain swap in the C-terminal regulatory domain. This entanglement suggests that FbpC is unable to open to the same extent as the homologous protein MalK. Using molecular dynamics we demonstrate that this is not the case: both NBDs open rapidly once ATP is removed. We conclude from this result that the closed structures of FbpC and MalK have higher free energies than their respective open states. This result has important implications for our understanding of the mechanism of power generation in ABC transporters, because the unwinding of this free energy ensures that the opening of these two NBDs is also powered.

## Introduction

Active transport is carried out within the cell by a class of integral membrane proteins called ATP-binding cassette (ABC) transporters. ABC transporters form one of the largest transporter gene families (Linton and Higgins, 1998) and use ATP to power the movement of a wide range of substrates across the membranes of both prokaryotic and eukaryotic cells. Not all the members of this family behave in this way: both the sulphonyl urea receptor (Aittoniemi et al., 2009) and the cystic fibrosis transmembrane regulator (Gadsby et al., 2006) use ATP to open an ion-conducting channel.

In the past few years, several high-resolution structures of ABC transporters have been determined by X-ray crystallography (Hollenstein et al., 2007; Oldham et al., 2008; Rees et al., 2009). The structure of mouse P-glycoprotein, one of the most studied ABC transporters, has also recently been published (Aller et al., 2009). These structures all have two transmembrane domains (TMDs) and two nucleotide-binding domains (NBDs—also known as ATP binding cassettes, ABCs). The TMDs bind the transported substrate and the NBDs provide the energy necessary for transport by binding and hydrolyzing two (or sometimes just one) molecules of ATP. Two ABC transporters, MalFGK_2_ and MsbA, have been captured in multiple states (Khare et al., 2009; Oldham et al., 2007; Ward et al., 2007). We could also compare the structures of unrelated ABC transporters in different states, for example ModBC (Gerber et al., 2008) and HI1470/1 (Pinkett et al., 2007). In these structures, the substrate-binding site is open to different sides of the membrane, confirming that ABC transporters function using an alternating access model (Jardetzky, 1966). It is usually assumed that energy is required to reorient the TMDs when the substrate is bound, and that this is provided by the NBDs. By analogy with internal combustion engines, this is often termed the “power-stroke” of transport (Smith et al., 2002).

The details of how the binding and hydrolysis of ATP leads to transport of the substrate is not yet known. Because NBDs from different ABC transporters share a high degree of sequence and structural homology (Oswald et al., 2006), it is generally assumed that there is a universal mechanism. Proposed mechanisms are usually categorized as either sequential or alternating (not to be confused with alternating access). This division is rarely clearly defined in the literature. We shall therefore define an alternating mechanism as one where only one molecule of ATP is hydrolyzed at a time (Jones and George, 2009; Oldham et al., 2008; Sauna and Ambudkar, 2007). One of these hydrolysis events might drive the power stroke. We shall further define a sequential mechanism as one where the binding of ATP drives the power stroke (Higgins and Linton, 2004; Janas et al., 2003; Linton and Higgins, 2007; Moody et al., 2002; Oldham et al., 2008). In this instance ATP hydrolysis ensures irreversibility. These definitions are neither mutually exclusive nor exhaustive, which makes determining and discussing the precise mechanism difficult. The NBDs of MalFGK_2_ and MsbA are “closed” and have ATP bound when the substrate-binding site is exposed to one side of the membrane, and are “open” and have no nucleotide bound when exposed to the other. In addition, no structures of an NBD with only one ATP molecule bound, either in isolation or as part of a full transporter, have been resolved. The available structural data therefore favor a sequential mechanism, although that does not rule out the possibility that ATP is also hydrolyzed alternately. It is also still possible that different ABC transporters use different mechanisms.

In this paper, we shall present the structure of FbpC, the nucleotide-binding domain of the *N. gonorrhoeae* iron-uptake ABC transporter, FbpABC. This is the first structure of a native NBD dimer captured in the prehydrolysis state with both ATP and a divalent metal ion bound. FbpC also has a hitherto unseen domain swap between two regulatory domains present at the C terminus of each NBD. The domain swap creates an unusually extended dimer interface, and the subsequent entanglement of the two monomers would appear to suggest that FbpC cannot open as far or as readily as MalK. We ran a series of detailed molecular dynamics simulations of both FbpC and Malk that demonstrate this hypothesis is false. Not only can FbpC open when ATP is removed, but also the speed at which this happens indicates that the closed structures of both NBDs have a higher free energy than their open states. Significantly, this result implies that not only is the closing of these NBDs powered (through the binding of ATP), but also the opening stroke is powered; some of the ATP-binding free energy is stored and then released following hydrolysis.

## Results

### The Structure of FbpC, a Domain-Swapped Nucleotide-Binding Domain Dimer

**Figure 1:**
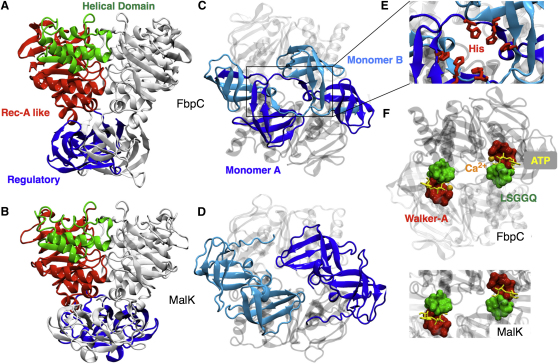
The Structure of FbpC, the Nucleotide-Binding Domain of the Iron Transporter, FbpABC, from *N. gonorrhoeae* (A) A side view of FbpC with the helical, Rec-A-like and regulatory domains colored green, red, and blue, respectively. (B) Side view of MalK for comparison (PDB:1Q12) (Chen et al., 2003). (C) The regulatory domains of FbpC as seen from below showing the domain swap. The domain from each monomer is colored dark and light blue. (D) The regulatory domains of MalK for comparison. (E) The six-histidine cluster at the base of the regulatory domain swap. (F) A simple comparison of how ATP binds to both FbpC and MalK. The Walker-A and LSGGQ motifs are colored red and green, respectively. ATP is colored yellow and the calcium ion, orange. A more detailed depiction of how ATP binds can be found in Supplemental Data.

We have solved the structure of the native nucleotide-binding domain, FbpC, from the *N. gonorrhoeae* iron-uptake ABC transporter, FbpABC (Lau et al., 2004). FbpC consists of 352 amino acids and the structure shows a domain-swapped physiological dimer in the asymmetric unit cell (Smith et al., 2002) (Figures 1A and 1C). This is the first observation to date of three-dimensional domain swapping in ABC importers. The crystal structure was determined to a final resolution of 1.9 Å and refined to a working R factor of 18.6% and R_free_ of 23.4% (Table 1).

The first 240 amino acids of each FbpC monomer show the same overall ATPase topology and sequence motifs previously observed for other NBD structures from ABC transporters (Oswald et al., 2006) (Figure 1A). The two ATP molecules are sandwiched between the Walker A and B motifs of the *cis*-monomer and the LSGGQ motif (C-loop) of the opposing *trans*-monomer (shown schematically in Figures 1F) as seen in other ATP-bound NBD structures (Oswald et al., 2006). A notable feature of the FbpC structure is that it is trapped in the ATP-bound prehydrolysis state with a divalent metal ion cofactor, even though the protein is an active ATPase (Lau et al., 2004). A divalent metal ion is found coordinated between the β- and γ-phosphates of each ATP and the Q-loop glutamine (Gln^87^) (see Figure S1 available online) in the electron density maps of FbpC. Given the high concentration of calcium in the crystallization conditions (0.2 M), it is highly probable that the ions observed in the active site of this structure are calcium. The metal ion cofactor is therefore directly linking the γ-phosphate of the ATP molecule to the “sensing” glutamine of the Q-loop rather than being indirectly linked via a water molecule. Structural alignments to other ATP bound NBD structures reveal that the majority of the residues involved in the binding and hydrolysis of ATP are in similar conformations (Figure S1). Interestingly, two are very different: the active-site glutamate, Glu^164^, which immediately follows the Walker B aspartate, and the H-loop histidine, His^197^. The H-loop histidine interacts with the γ-phosphate of ATP in both MalK (Chen et al., 2003) and MJ0796 (Smith et al., 2002). Based on studies of HlyB, the NBD of the Hemolysin B transporter, Zaitseva and colleagues proposed that the H-loop histidine stabilizes the transition state of the ATP reaction and mediates communication between the monomers through interactions with the D-loop of the opposing monomer (Zaitseva et al., 2005a). The role of the conserved glutamate is unclear; it has been proposed to act both as the catalytic base in ATP hydrolysis (Moody et al., 2002) as well as in concert with the H-loop histidine in a substrate-assisted catalysis model of ATP hydrolysis (Zaitseva et al., 2005a). A striking feature of the FbpC active site is that neither of these residues makes any interaction with each other or the γ-phosphate of ATP (Figure S1). This suggests that the presence of calcium in the cofactor-binding site may have caused the catalytic dyad of His^197^ and Glu^164^ to adopt an unproductive conformation, although His^197^ still makes the *trans* interactions to the carbonyl oxygens of Ser^167^ and Leu^169^ on the D-loop of the opposing monomer as observed in HlyB. This effect of calcium on the conformation of the His-Glu catalytic dyad was unexpected, and further work is being carried out to investigate the implications of this observation.

FbpC also contains an additional C-terminal regulatory domain of ∼110 amino acids (RD; residues 242–352) that adopts two OB-folds (Arcus, 2002) per monomer. These are similar in topology to those seen in the NBD from the maltose uptake ABC transporter, MalK (Chen et al., 2003; Diederichs et al., 2000) (Figures 1C and 1D). Unlike MalK, the domains in FbpC undergo a substantial domain swap between the two monomers. The peptide chain immediately preceding the N-terminal ATPase domain of each monomer initially forms the first OB-fold before making the first β strand of the second OB-fold on the same side of the dimer. Finally, the peptide chain swaps over and forms the bulk of the second OB-fold on the other side of the dimer (Figures 1C and 1D). This is the first observation of such a domain swap in the structure of an ABC importer.

A small cavity is formed at the base of the dimer by the peptide chains as they cross over to form the remaining OB-folds. Three histidines from each monomer form a well-ordered cluster of six histidines (Figure 1E). Two of the six imidazole side chains of the cluster orientate toward the center of the cavity, suggesting this cluster might be a possible iron requisition store. Recent studies on both a methionine uptake ABC transporter (Kadaba et al., 2008) and a molybdate/tungstate ABC transporter (Gerber et al., 2008) have established that the substrates of ABC transporters can act as allosteric regulators through binding to the NBD dimer (Rees et al., 2009). The function of this histidine cluster in FbpC is unknown, although because the use of histidines in coordinating the binding of iron and other metals is well established, a role in iron binding seems plausible.

### FbpC Does Not Open as Quickly as MalK

**Figure 2:**
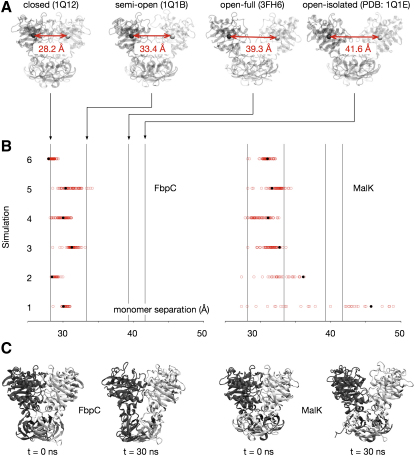
The Distributions of Monomer Separations for FbpC and MalK (A) The monomer separation is defined as the distance between the center of mass of each monomer (excluding the Regulatory Domain). Shown in red are the distances for the four reference crystal structures of MalK. (B) The distributions of the monomer separations measured from the six 30 ns simulations of FbpC and MalK. The last datapoint is colored black. (C) Illustrative snapshots taken from the beginning and end of one of the simulations for each NBD. The monomers are colored dark and light gray.

**Figure 3:**
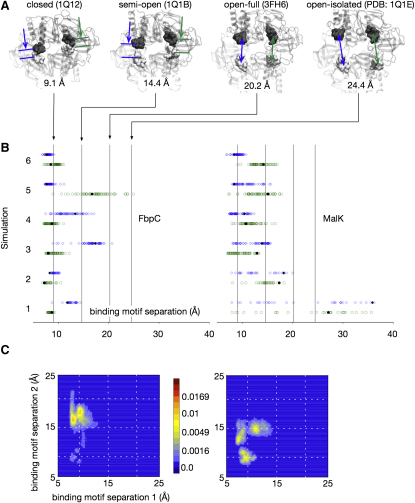
The Distributions of Binding Motif Separations for FbpC and MalK (A) The binding motif separation is defined as the distance between the center of masses of the Walker-A and LSGGQ motifs on opposing monomers. Shown in blue and green are the two distances for each of the four reference MalK structures. (B) The distributions of the binding motif separations, in blue and green, measured from the six apo 30 ns simulations of FbpC and MalK. (C) Plotting the two binding motif separation from each simulation against the other yields a probability density plot. Shown here are two examples; the others can be found in Supplemental Data. The plots demonstrate the stochastic and discrete behavior of both NBDs. The dashed white lines are, in order of increasing separation, the binding motif separations for the closed, semiopen, and open-full MalK reference structures.

To test the hypothesis that, due to its entangled regulatory domain, FbpC does not open as far or as readily as MalK, we shall take ATP-bound closed structures of both NBDs, remove both molecules of ATP, and then simulate the subsequent dynamics. We shall discuss the reasons for choosing to study an unphysiological step later. From each simulation we measured two distances. The first is simply the distance between the centers of mass of the heavy atoms of the Rec-A like and helical domains (i.e., excluding the regulatory domain) of the two monomers (Figure 2A). We shall call this the *monomer separation*. This gives a simple measure of how far each NBD has opened. The monomer separation cannot distinguish between a uniform opening of the monomers and an asymmetric motion where, for example, one of the two NBD interfacial binding sites has opened significantly but the other has remained shut. To differentiate between these two cases, we also measured the distance between the centers of mass of the heavy atoms of the Walker-A and LSGGQ sequences of each ATP-binding site (Figure 3A). These motifs belong to different monomers, and therefore the distance between them will report the opening of the interfacial ATP binding site. We shall call this distance the *binding interface separation*.

We put the magnitudes of the distances in context by comparing the results of the simulations to the same distances measured on experimentally determined structures of MalK. These are the closed, semi-open, and open structures of isolated MalK dimers determined by Chen and colleagues, where each has ATP, ATP and nothing bound, respectively (Chen et al., 2003). There are also two structures of MalK with ADP bound (Lu et al., 2005) that are open to approximately the same degree as the semi-open structure, but we will not use this structure in the analysis because the distances vary significantly between the dimers in the crystal unit cell. As noted, there are also two structures of MalFGK_2_. The distances measured from the outward-facing structure where the MalK dimer is closed and ATP bound (Oldham et al., 2007) are within 0.2 Å of the closed isolated MalK dimer and are therefore almost identical. However, distances measured from the recent inward-facing structure of MalFGK_2_, where the MalK dimer is open and no ATP is bound, were included (Khare et al., 2009). These distances are intermediate between those measured from the semiopen and open isolated MalK structures, no doubt due to the interaction with the TM domain subunits. These structures are designated in ascending order of separation (closed, semiopen, open-full, and open-isolated) and they are shown schematically in Figures 2A and 3A.

As a control we first ran two simulations each of FbpC and MalK with ATP bound (Figure S2). For both NBDs the monomer and binding interface separations remained between that of the closed and semiopen MalK structures, indicating that these structures are stable during the simulations. Both the MalK monomer and binding motif separations have larger fluctuations than FbpC, suggesting that ATP-bound MalK is less stable than ATP-bound FbpC. We then removed both molecules of ATP from the experimental structures of FbpC and MalK and ran six further simulations of each NBD. Multiple simulations were run to increase the likelihood that we could distinguish any differences in the dynamics of the two NBDs. We ran four of these simulations using one forcefield (GROMOS53a6) and two using another (CHARMM27). This necessitated that each set of simulations was run using a different molecular dynamics package (see Experimental Procedures). Our conclusions are therefore robust.

Only in two of the six simulations did FbpC open as far as the semiopen MalK structure as measured by the monomer separation (Figure 2). MalK, however, opened this far in four simulations. The two simulations of MalK that did not open this far had still opened further than the remaining four simulations of FbpC, suggesting that MalK opens more readily. This trend can also be seen in the binding motif separation distributions (Figure 3). Four of the twelve binding interfaces of FbpC have separated at least as far as the semiopen MalK structure, with two opening as far as the open-full structure and only one as far as the open-isolated structure. Nine of the twelve binding interfaces of MalK have separated at least as far as the semiopen MalK structure, with three opening as far as the open-full structure and two as far as the open-isolated structure. Interestingly, the NBDs are not opening symmetrically. For example, the monomer separation measured from simulations 1 and 4 of FbpC suggests that the NBD has not opened significantly, yet one of the two binding motif distances is equal to or greater than the semiopen MalK structure. Both NBDs are therefore opening asymmetrically, and this can be seen more clearly in the 2D probability density distributions for both binding motif separations for each NBD dimer (Figures 3 and S3). These plots also show that the opening dynamics, when viewed over this short timescale at least, are not smooth but discrete. This behavior might, of course, appear different when viewed over much longer timescales.

To test if the opening of each binding interface is independent of the other, we used the randomization test for independence (see Experimental Procedures). Using this test, the probabilities of the binding interface openings being independent are 0.61 and 0.17 for FbpC and MalK. This suggests, but does not prove that, as shown by experiment for MalK (Davidson et al., 1996), each binding interface is not independent of the other. As already noted, the differences in the distributions of both distances (Figures 2 and 3) suggest that MalK opens more readily than FbpC. We can check this using the two-sample Kolmogorov-Smirnov statistical test to investigate whether the two data sets made up the final or average monomer separations and the final or average binding motif separations for each NBD differ significantly. The probabilities of all these data sets being drawn from the same distribution are 0.01, 0.08, 0.02, and 0.07, respectively, confirming our observation that FbpC has not opened as far or as rapidly as MalK in the simulations. The first simulation of MalK was identified as an outlier by this process and was therefore excluded from all subsequent analysis. This does not impact our earlier analysis of independence. Finally, we note that, regardless of the difference above, both these NBDs open surprisingly quickly. For example, for the monomers to separate as far as the semiopen MalK structure, the centers of masses must move apart by 5.2 Å in less than 30 ns.

### Each NBD Monomer Is Charged

**Figure 4:**
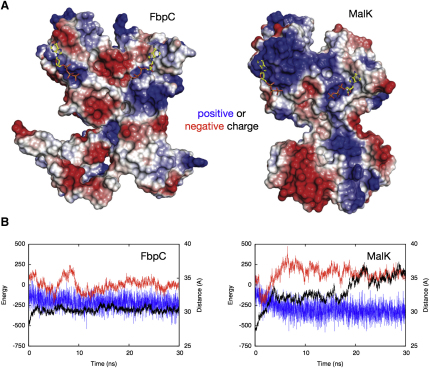
It Is Not Known What Forces Are Responsible for the Opening of Both NBDs (A) Both NBDs have significant electrostatic charges on their surfaces. Positive and negative charges are drawn in blue and red and ATP is drawn in yellow. Both structures are presented with the active site facing out of the page. The surfaces were calculated using the APBS plugin (Baker et al., 2001) of PyMol (DeLano, 2008). (B) The monomer separation (in black), internal bonded energy of the dimer (in blue), and nonbonded interaction energy (in red) between the monomers are all plotted on a single graph to check for correlations. Only two examples are shown; the remaining plots are in Supplemental Data.

This raises the question: what forces are responsible for driving the NBDs apart? At least three potential sources can be identified: (1) an electrostatic repulsion between the two NBD monomers (Moody et al., 2002; Smith et al., 2002), (2) the relaxation of strain in the closed structure (Zaitseva et al., 2006), or (3) an increase in solvation energy as the accessible surface area of the protein increases. As observed for the MJ0796 NBD (Moody et al., 2002; Smith et al., 2002), there are significant areas of positive and negative charge distribution at the interface between the monomers of both NBDs (Figure 4A), supporting the electrostatic repulsion hypothesis. If the electrostatic interaction energy between the two monomers decreased as the monomer separation increases, this would corroborate the hypothesis, as has been observed in other cases (Zachariae and Grubmuller, 2006). Alternatively, a correlation between the internal energy stored in the bonds of the two monomers with the monomer separation would support the second hypothesis. There are instances (e.g., Figure 4B) where the internal bonded energy appears negatively correlated with the monomer separation, but this is not repeated in the other simulations (Figures 4B and S4). We conclude that either more than one type of force is responsible for driving the monomers apart, or our approach is unable to resolve the relevant correlations. This might be because the fluctuations in the energies are large due to the thermal motion of the protein, or the simple distances we measure might hide other motions of the dimer that reduce either the electrostatic interaction energy or the bonded internal energy. The effect could be very subtle: simple calculation shows that a single net charged residue could provide sufficient force to drive the monomers apart.

## Discussion

We have shown that FbpC does not open as far as MalK when ATP is removed from their respective closed structures. This difference we infer is due to the substantial domain swap in the regulatory domain of FbpC. We have deliberately not simulated a physiological transition because removing ATP from a closed structure and studying the subsequent dynamics yields information about the relative energies of the open and closed states. Because both NBDs open very rapidly once ATP is removed, the closed structures of FbpC and MalK have higher free energies than their open states. Alternatively, we could say that the closed structures of both NBDs are “tense.” Because this conclusion is a thermodynamic property of the structures of FbpC and MalK, it is independent of the details of exactly how ATP is hydrolyzed, or how the NBDs transmit this motion to the TMDs; whatever the details, this difference in free energy will still be present. Because ATP mediates many of the interactions between the two monomers in the closed state, it is often described as “gluing” the two monomers of a NBD together (Karpowich et al., 2001; Zaitseva et al., 2005b). Our simulations show that ATP is holding them closed, and so this metaphor is doubly appropriate for MalK and FbpC.

**Figure 5:**
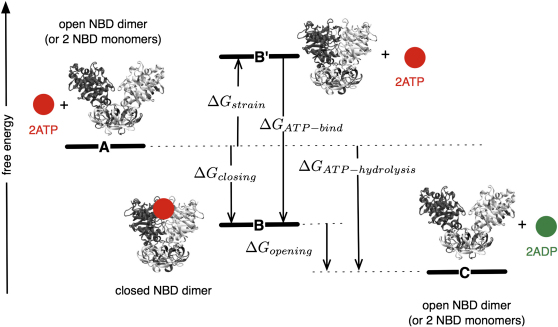
The Closed Structures of MalK and FbpC Are Tense Our simulations studied the effect of removing ATP from the closed, ATP-bound structure. This is the unphysiological B′ to A transition. Because both NBDs open rapidly, the free energy of state B' must be greater than that of state A, which we attribute to “strain energy.” The closing stroke of transport is the A to B transition. The difference in free energy between state B' and state B, where ATP is bound, is simply the binding free energy of ATP. Some of this energy is therefore stored as the strain energy when ATP binds. The opening stroke of transport is the B to C transition, which is therefore driven partly by the hydrolysis of ATP and partly by the unwinding of the strain energy built up during the closing stroke. ATP and ADP are drawn as red and green circles, respectively. Increasing free energy is plotted along the *y* axis.

As described earlier, the available structural evidence is consistent with sequential mechanisms, such as the ATP switch model (Higgins and Linton, 2004; Linton and Higgins, 2007). In these models, the binding of ATP is assumed to drive the reorganization of the TMDs, thereby leading to transport (the power stroke). Our results show that, for these NBDs at least, part of the binding free energy of ATP is stored (somehow) as a “strain energy” during the power-stroke, and this energy is released following hydrolysis. This is shown schematically in Figure 5. We have been unable to determine what is driving the monomers apart. It is, of course, not certain that all NBDs behave as we have described, and our conclusions therefore might not apply to all ABC transporters.

It is not known in detail how the free energy made available when ATP is hydrolyzed is converted into mechanical work that can be used to move the TMDs. It cannot be via heat because this would be inefficient. The confined phosphate must more directly exert a force on the NBD (MacKay and MacKay, 2006). Likewise, it is not known when or the order in which the hydrolysis products unbind from the NBD. Whatever the mechanism, the additional strain energy stored in the tense closed NBD structure will reduce the time taken for the NBD to reset the TMDs, and this might help improve the quality of the switch. It has been shown directly by computer simulation (Oloo et al., 2006) and indirectly by X-ray crystallography (Lu et al., 2005) that MalK closes rapidly around ATP. The binding free energy of ATP is therefore large enough to close the MalK dimer and build up a strain energy, which, as shown in this study, is large enough to rapidly open the NBD when ATP is removed. This is analogous to pinching a simple spring shut: our fingers (the binding of ATP − ΔG_ATP-bind_) are strong enough to compress the spring, but the strength of the spring (the strain energy − ΔG_strain_) is large enough that, when we let go, the spring quickly returns to its original length. Of course, when coupled to the TMDs, these motions will be far slower, but that does not change any of the conclusions about the ability of the NBDs to do work on the TMDs as the NBDs open. Ultimately, one must also consider the ability of the TMDs to do work on the NBDs. For example, Oldham et al. (2008) propose that the NBD dimer interface is held open by the TMDs; whether this is true depends on if the mechanics of the TMDs or the NBDs dominate. We caution that it is not possible to draw any conclusions about whether the asymmetrical opening observed in our simulations would persist over much longer timescales. This is because none of the structures simulated here have reached equilibrium. Although it is tempting to conclude from this type of observation that an alternating mechanism is more likely (Jones and George, 2009), one would have to run simulations for long enough to allow the structure to fully relax.

Not only should we keep in mind that there might be no single mechanism to describe ABC transport, but we should also assess whether it is useful to characterize the possible mechanisms as either sequential or alternating. The simulations in this study strongly suggest that the opening of the interfaces is stochastic and discrete. Our results are consistent with the proposal that NBDs behave stochastically (Campbell and Sansom, 2005; Wen and Tajkhorshid, 2008). For some ABC transporters, the binding of a single molecule of ATP might be sufficient to rearrange the TMDs and drive transport. The relative kinetics of ATP binding, dimer closure, and TMD rearrangement would determine whether one or two molecules of ATP would bind per transport cycle. This model therefore explains both the noninteger and variable ATP stoichiometry often measured for ABC transporters (Davidson and Nikaido, 1990).

Each simulation of the dynamics of FbpC and MalK is different, and therefore we must rely on statistical tests to distinguish any differences in behavior. More simulations would reduce our statistical errors and strengthen our conclusions. As mentioned above, the simulations only capture the very first steps of the opening, so much longer simulations might allow us to observe a degree of convergence in the behavior of all the simulations. This might also permit conclusions to be drawn about whether the open state is best represented by a single structure or by an ensemble of structures. One still faces considerable biochemical obstacles to be overcome when studying ABC transporters. For example, in theory one could test whether electrostatics drives the opening of FbpC by mutating charged residues and measuring the difference in transport. In practice, the difficulty in accurately manipulating the electrostatic surface of protein macromolecules makes this type of experiment difficult to perform accurately. Membrane transporters are inherently dynamic, and a deeper understanding of their function is difficult to ascertain from static crystallographic snapshots alone. Combined approaches where in silico techniques are used to study the dynamics of structural snapshots determined by X-ray crystallography will provide valuable new insights into the mechanism of these proteins. We have demonstrated in this study that that the closed structures of two NBDs of ABC transports, FbpC and MalK, are tense compared with their open states, that this strain energy is built up when ATP binds and that it has the potential to force the NBDs apart following the hydrolysis of ATP.

## Experimental Procedures

### FbpC Cloning and Purification

The gene encoding the FbpC protein was amplified from a clinical strain of *Neisseria gonorrhoeae* genomic DNA (from the Scottish *N. gonorrhoeae* Reference Laboratory) and cloned as an NcoI-XhoI PCR fragment into the T7 expression vector pET28a to give the plasmid pET-28a/FbpC/Ng encoding a C-terminal 6 His-tag. The sequence showed that the encoded FbpC contains two changes (V147A and F283Y) to the FbpC available on the *N. gonorrhoeae* genome (GenBank accession code AE004969). These residues do not lie within areas of importance in the structure of the enzyme. The strain BL21 (DE3) carrying pET-28a/FbpC/Ng was induced at OD_600_ 0.8 by addition of 0.2 mM IPTG and growth continued for a further 3 hr at 25°C. The FbpC-expressing pellet was resuspended in 25 mM Tris buffer (pH 8.0) containing ATP (5 mM), EDTA (0.1 mM), 20% glycerol. The resuspended pellet was lysed by sonication and centrifuged at 25,000 x *g* for 30 min at 4°C. FbpC was purified using a two-step procedure. The protein was initially purified using Ni-NTA silica (Promega). Five milliliters Ni-NTA silica beads were incubated with the lysate for 1 hr at 4°C with the addition of 20 mM imidazole. The resin was then packed into a gravity flow column and washed in 30 column volumes of 25 mM Tris buffer (pH 8.0) containing ATP (5 mM), NaCl (0.5 M), 20% glycerol, imidazole (0.05 M). FbpC was eluted using a step gradient to 0.25 M imidazole. The eluted FbpC was then diluted 1/10 in buffer containing no imidazole and the protein incubated with 5 ml TALON cobalt-affinity resin (BD Biosciences) for a further 1 hr at 4°C. The protein was washed and eluted as described above, except the imidazole concentration was lowered to 30 mM during the wash step. The purity of the eluted protein was checked by SDS-PAGE and was >99% pure. Purification of SeMet labeled FbpC was identical except that all buffers also contained 0.5–1 mM tris(carboxyethyl)phosphine (TCEP) as a reducing agent to avoid oxidation of incorporated selenomethionine residues.

### Selenomethionine Labeling of FbpC

Strain BL21 (DE3) carrying pET-28a/FbpC/Ng was grown overnight in MDG media (Studier, 2005). The overnight cultures were used to inoculate 5 l M9 media supplemented with magnesium sulfate, glucose, thiamine, biotin, and trace metals. The cultures were grown to an OD_600_ 0.8 at 25°C and further supplemented with 100 mg l^−1^ lysine, threonine, phenylalanine, and 50 mg l^−1^ leucine, isoleucine, valine, and selenomethionine were added to promote shutdown of methionine biosynthesis and selenomethionine uptake. FbpC expression was induced after 15 min by addition of 0.2 mM IPTG and the cultures grown for a further 3 hr at 25°C.

### Crystallization and Data Collection

FbpC stock (5 mg ml^−1^ FbpC, 5 mM ATP in buffer containing 10 mM HEPES [pH 7.50], 2 mM EDTA, 20% glycerol) was initially screened using the Mosquito nano drop liquid handler against the sparse matrix screens JCSG and PACT. Initial crystals were observed in condition 23 of the PACT crystal screen (QIAGEN) at 19°C. This condition was then optimized using a grid and the final crystals obtained in 20% PEG 6000, 0.1 M MES (pH 6.50), 0.2 M calcium chloride using a 1 μl:1 μl drop ratio in sitting drop crystallization plates (Innovadyne). Crystals grew after 2 days at 19°C. Suitable crystals were transferred briefly to mother liquor plus 20% glycerol for cryoprotection before flash cooling in liquid nitrogen. Native data were collected on beamline ID23eh1 at the ESRF and selenomethionine MAD data collected on beamline 10.1 at the SRS (Daresbury, UK).

### Structure Determination

The MAD data were integrated, indexed and reduced using HKL-2000 (Minor et al., 2006). The native dataset was integrated, indexed and reduced using MOSLFM (Leslie, 1992) and SCALA from the CCP4 suite (CCP4, 1994). Phases were obtained using MAD phasing with the program SOLVE (Terwilliger and Berendzen, 1999), which gave an overall figure of merit of 0.40 and a Z-score of 41.5 with six selenium sites. The data were then merged with the native data using all data to a resolution of 1.9 A. The experimental and native data were merged and density modification, with phase extension to 1.9 Å, was performed in RESOLVE (Terwilliger, 2000, 2003). The resulting phases allowed the program to build approximately 90% of the structure using the model building algorithm. Subsequent rounds of model building, in O and coot (Emsley and Cowtan, 2004) and refinement in Refmac 5 (Murshudov et al., 1997), gave the final structure, which was assessed for quality using MolProbity (Davis et al., 2007). The coordinates and associated structure factors have been deposited with the Protein Data Bank (PDB, accession number 3FVQ).

### Molecular Dynamics

A total of eight FbpC and eight MalK simulations were run; each had a duration of 30 ns, making 0.48 μs in total. For each NBD dimer, two simulations were run with ATP bound and six were run after ATP had been removed. The ATP-bound simulations used the GROMOS53a6 forcefield (Oostenbrink et al., 2004) and were run using GROMACS3.3.1 (van der Spoel et al., 2005). Four of the ATP-free simulations were run in the same way. The remaining two ATP-free simulations used the CHARMM27 (Foloppe and MacKerell, 2000) forcefield and were run using NAMD2.6 (Phillips et al., 2005).

For MalK we used the PDB:1Q12 structure (Chen et al., 2003). The distance between the conserved (sensing) glutamine and the γ-phosphate varies significantly between the four monomers contained in the PDB file. We assumed that this residue is in contact with the γ-phosphate in the closed, ATP-bound state (as it is in FbpC) and constructed a symmetrical dimer from chain C of the PDB:1Q12 structure. This approach has been used in studies of other NBDs (Jones and George, 2007). A magnesium ion was added by fitting the resulting dimer onto another NBD structure. The crystal waters were unevenly distributed among the four monomers and so these were not included. Because the structure of FbpC is symmetric, chains A and B were used and the crystallographic waters and bound ATP and calcium ions were included. Each dimer was then rotated to minimize the number of water atoms required to ensure that the minimum distance from the protein to the edge of the box was 10 Å. The sizes of the boxes were 92.4 × 94.9 × 88.6 Å for FbpC and 90.0 × 85.7 × 90.0 Å for MalK. The total numbers of atoms were 68,041 and 59,561 for the ATP/ATP-bound simulations of FbpC and MalK, respectively. NaCl was added to neutralize the charge of the system. Apo complexes were constructed by removing both molecules of ATP.

The energy of each simulation unit cell was minimized by the application of 1000 steps of the steepest descent algorithm. The temperature was then increased from 100 K to 310 K in steps of 20 K with 50 ps of molecular dynamics run at each temperature. The production trajectory of 30 ns was then run. PME (Darden et al., 1993) was used to calculate the electrostatic forces with a Fourier spacing of 1 Å and real space cutoffs of 12 and 13.5 Å for the GROMOS and CHARMM forcefields, respectively. For the GROMOS simulations, the van der Waals forces were not computed between any pair of atoms separated by more than 10 Å, with a switching function applied between 9 and 10 Å; the CHARMM simulations used a cutoff of 12 Å and applied a switching function between 10 and 12 Å. In all CHARMM simulations, SHAKE (Ryckaert et al., 1977) and SETTLE (Miyamoto and Kollman, 1992) were applied to constrain the lengths of all bonds that involve a hydrogen. In all GROMOS simulations, the lengths of all bonds were constrained using LINCS (Hess et al., 1997). This allowed an integration timestep of 2 fs to be used. The temperature was held at 310 K using Langevin dynamics with a damping coefficient of 1 ps^−1^. A Berendsen barostat was applied anisotropically with a compressibility of 4.5 × 10^−5^ bar^−1^ and relaxation times of 200 fs and 1 ps for the GROMOS and CHARMM simulations, respectively (Berendsen et al., 1984). Finally, frames were written to disc every 1 ps.

### Statistical Analyses

We used the randomization test for independence rather than more conventional tests (such as the chi-square test) because the sample size is small. We arbitrarily classify binding interface as open if it its measured binding motif separation is larger at any time during the simulation than the same distance measured from the semiopen structure of MalK. From this we count in how many simulations both, only one, or neither binding interface opened. The chi-square value of our observed data set is calculated. Then we numerically generate a large number of similar distributions, assuming the binding interfaces are independent (by calculating a probability of opening from our observations). The chi-square value of each of these is also calculated, and the probability that the binding interfaces are independent is the proportion of the randomly generated data sets that have a chi-square value greater than or equal to our observed data set. We used the two-sample Komologorov-Smirnov statistical test, although using the Mann-Whitney U test instead does not alter our conclusions. These tests assess whether the distributions of final or average distances are drawn from the same distribution. All these statistical analyses were coded in python using the numpy and scipy modules.

## Figures and Tables

**Table 1:** Data Collection and Refinement Statistics for *N. gonorrhoeae* FbpC

Data Collection and Phasing				
		Selenomethionine		
	Native	Peak	Inflection	Remote
Space group	P2_1_2_1_2_1_	*P*2_1_2_1_2_1_		
Cell dimensions: *a, b, c* (Å)	55.86, 89.09,149.01	55.9, 89.36, 149.98		
Beamline	ID23eh1 (ESRF)	SRS 10.1 (Daresbury)		
Wavelength	0.979	0.979	0.980	0.954
Resolution (Å)^a^	55.0-1.9 (2.0-1.9)	50.0-2.7 (2.8-2.7)	50.0-2.7 (2.8-2.7)	50.0-2.7 (2.8-2.7)
Total observations	210101	164698	99820	175583
Unique reflections	51975	21339	20187	23090
Redundancy	4.0 (3.4)	7.7 (5.4)	4.9 (3.0)	7.6 (5.2)
R_sym_ (%)^b^	0.104 (0.401)	0.149 (0.405)	0.133 (0.277)	0.116 (0.310)
*I/σI*	5.6 (1.8)	13.3 (3.7)	10.8 (3.00)	17.1 (4.6)
Completeness (%)	88.7 (92.1)	99.6 (96.3)	94.1 (69.6)	99.5 (96.7)
	Refinement (Refmac 5.2.0019)			
Resolution (Å)	20 – 1.9			
Number of reflections	49323			
R_cryst_^c^/ R_free_^d^ (%)	18.1 / 23.4			
Number of atoms				
Protein	5376			
Ligand/ion	66			
Water	540			
*B*-factors				
Protein	20			
Ligand/ion	18			
Water	26			
R.m.s. deviations				
Bond lengths (Å)	0.016			
Bond angles (°)	1.648			

a Values in parentheses are for the highest-resolution shell.

b R_sym_ = Σ|I– < I > | / I, where < I > is the average intensity over symmetry equivalent reflections.

c R_cryst_ = Σ|Fo– Fc| / ΣFo, where summation is over the data used for refinement.

d R_free_ was calculated using 5% of excluded data from refinement.

## Accession Numbers

Coordinates have been deposited in PDB with accession code 3FVQ.

## Supplemental Data

Document S1. Four Figures
