# Supplementary material for: Insights into How Nucleotide-Binding Domains Power ABC Transport
Source: Structure. 2009 Sep 9;17(9-2):1213–22. doi: 10.1016/j.str.2009.07.009 (PMC2896483; doi:10.1016/j.str.2009.07.009)
Supplement: Document S1. Four Figures [file mmc1.pdf]

Structure *17*

Supplemental Data

Insights into How Nucleotide-Binding Domains Power ABC Transport

Simon Newstead, Philip W. Fowler, Paul Bilton, Elisabeth P. Carpenter, Peter J. Sadler, Dominic J. Campopiano, Mark S. P. Sansom, and So Iwata

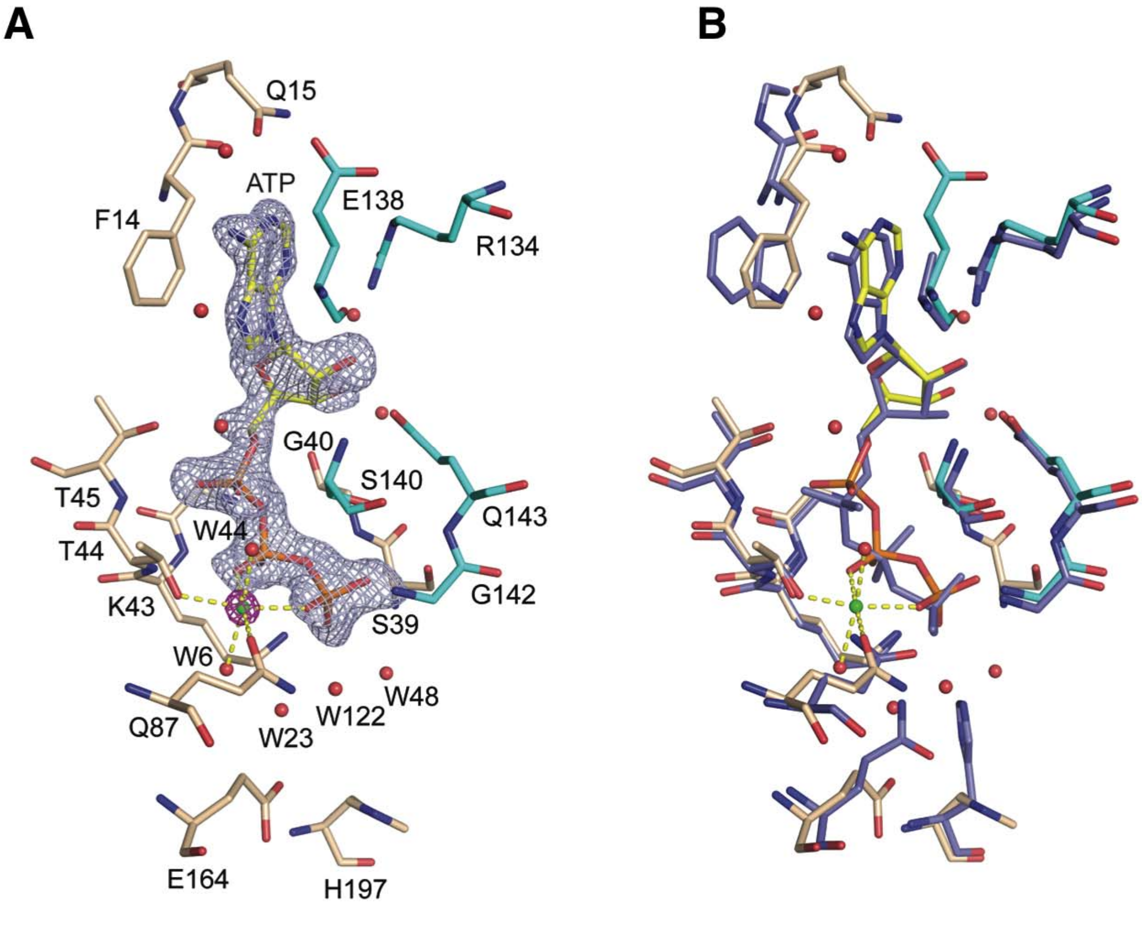

Figure S1. **The arrangement ATP and Ca^{2+} ions and key amino acid residues in one of the two active sites present in the FbpC dimer**

(A) F_o - F_c electron density omit maps (pale blue) are shown for ATP and the bound Ca^{2+} ion (magenta) along with key amino acids that form the binding pocket and active site from monomer A (wheat carbons) and momomer B (cyan carbons). Maps are contoured at 4 σ and 20 σ respectively. Waters are shown as red spheres.
(B) Superposition of the equivalent residues from E. coli MalK (PDB: 2r6g)

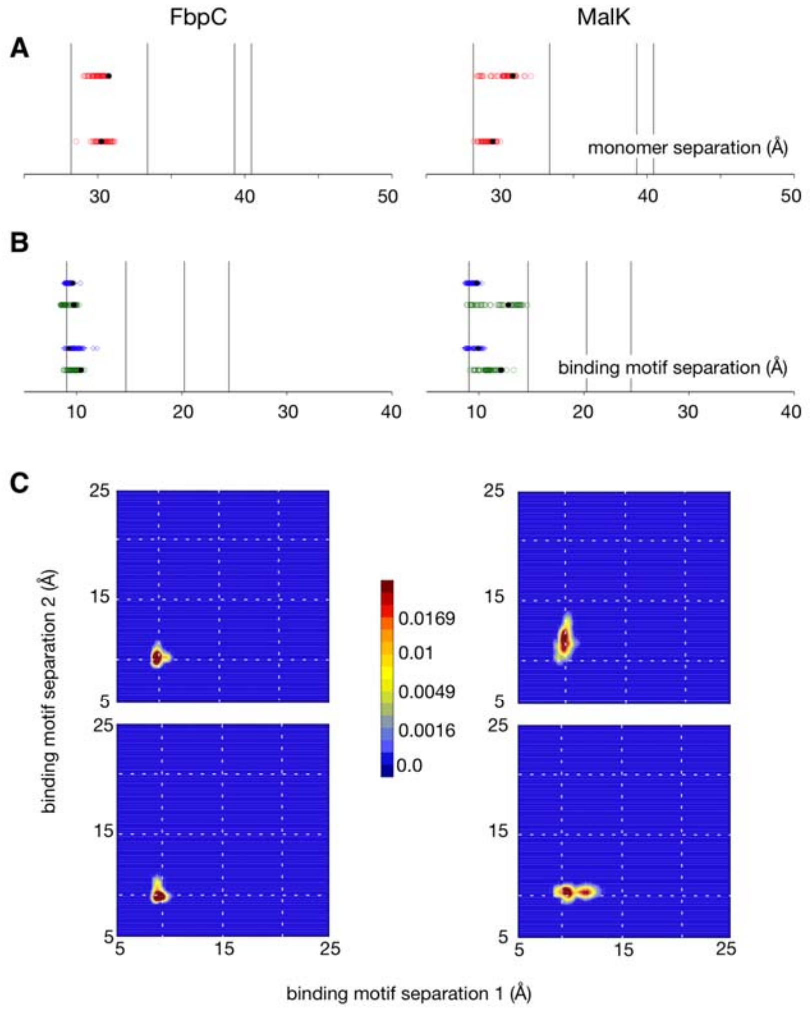

Figure S2. The binding interfaces of both NBDs remain closed when ATP is bound

(A) Plotted are the distributions of the monomer separations measured from the two ATP-bound 30 ns simulations of FbpC and MalK. The monomer separation is defined as the distance between the center of mass of each monomer (excluding the Regulatory Domain).

(B) The distributions of the binding motif separations, in blue and green, measured from the two ATP-bound 30 ns simulations of FbpC and MalK. The binding motif separation is defined as the distance between the center of masses of the Walker-A and LSGGQ motifs on opposing monomers.

(C) The four probability density plots made by plotting the two binding motif separations from each simulation against each other. The dashed white lines are, in order of increasing separation, the binding motif separations for the closed, semi-open (Chen et al., 2003) and open-full (Khare et al., 2009) MalK reference structures.

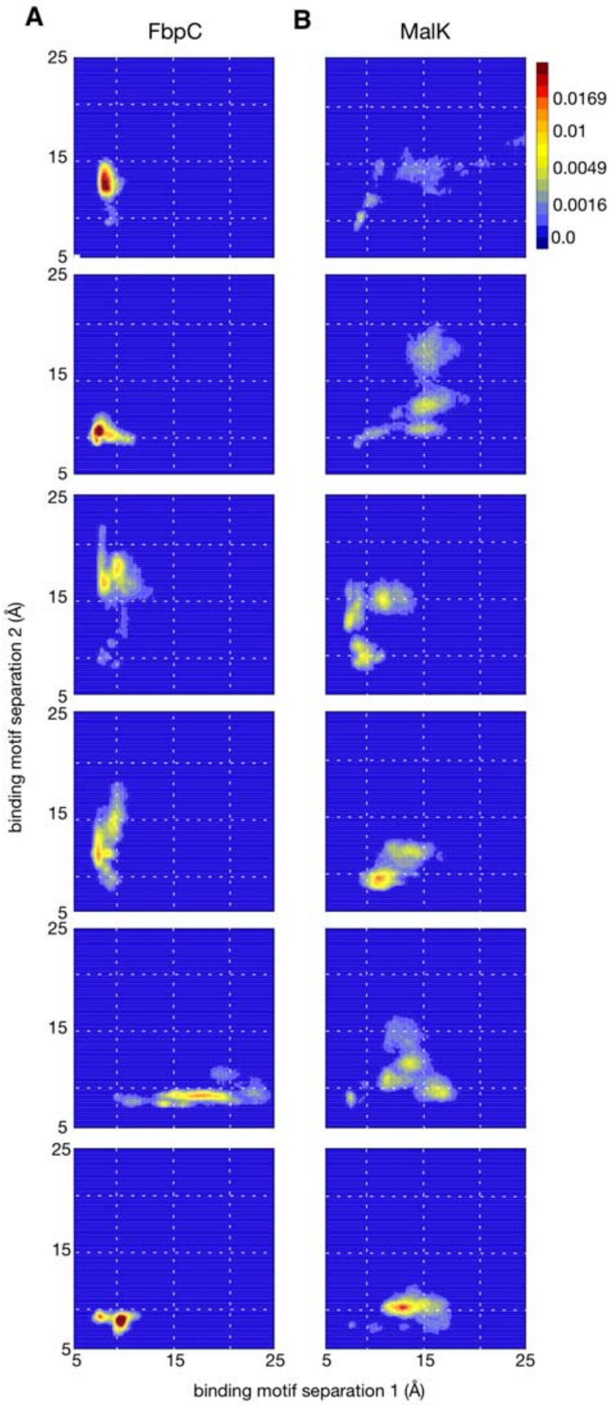

Figure S3.
The probability densities generated by plotting the two binding motif separations against one another for all six apo (A) FbpC and (B) MalK simulations. The dashed white lines are, in order of increasing separation, the binding motif separations for the closed, semi-open (Chen et al., 2003) and open-full (Khare et al., 2009) MalK reference structures.

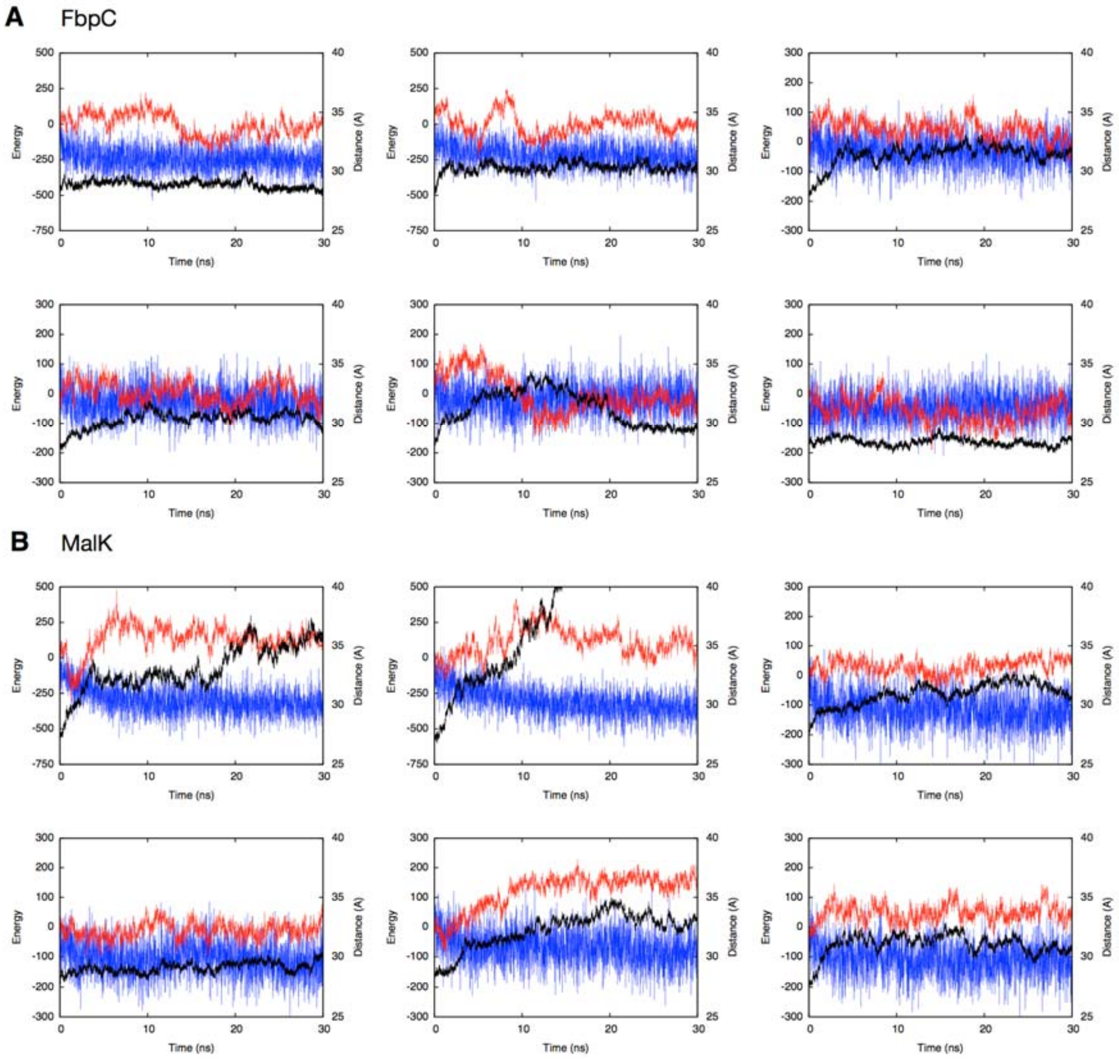

Figure S4.
The monomer separation (in black), internal bonded energy of the dimer (in blue) and nonbonded interaction energy (in red) between the monomers are all plotted on a single graph to check for correlations for (A) FbpC and (B) MalK. There is no clear correlation between the opening of the dimer and either of the energies.

SUPPLEMENTAL REFERENCES

Chen, J., Lu, G., Lin, J., Davidson, A.L., and Quiocho, F.A. (2003). A tweezers-like motion of the ATP-binding cassette dimer in an ABC transport cycle. Molecular Cell *12*, 651-661.

Khare, D., Oldham, M.L., Orelle, C., Davidson, A.L., and Chen, J. (2009). Alternating access in maltose transporter mediated by rigid-body rotations. Mol Cell *33*, 528-536.
